# Supplementary material for: Exponentially decreasing exposure of antigen generates anti-inflammatory T-cell responses
Source: bioRxiv. 2023 Sep 17:2023.09.15.558014. Preprint. [Version 1] doi: 10.1101/2023.09.15.558014 (PMC10516048; doi:10.1101/2023.09.15.558014)
Supplement: Supplement 1 [file media-1.pdf]

Supplementary Figures

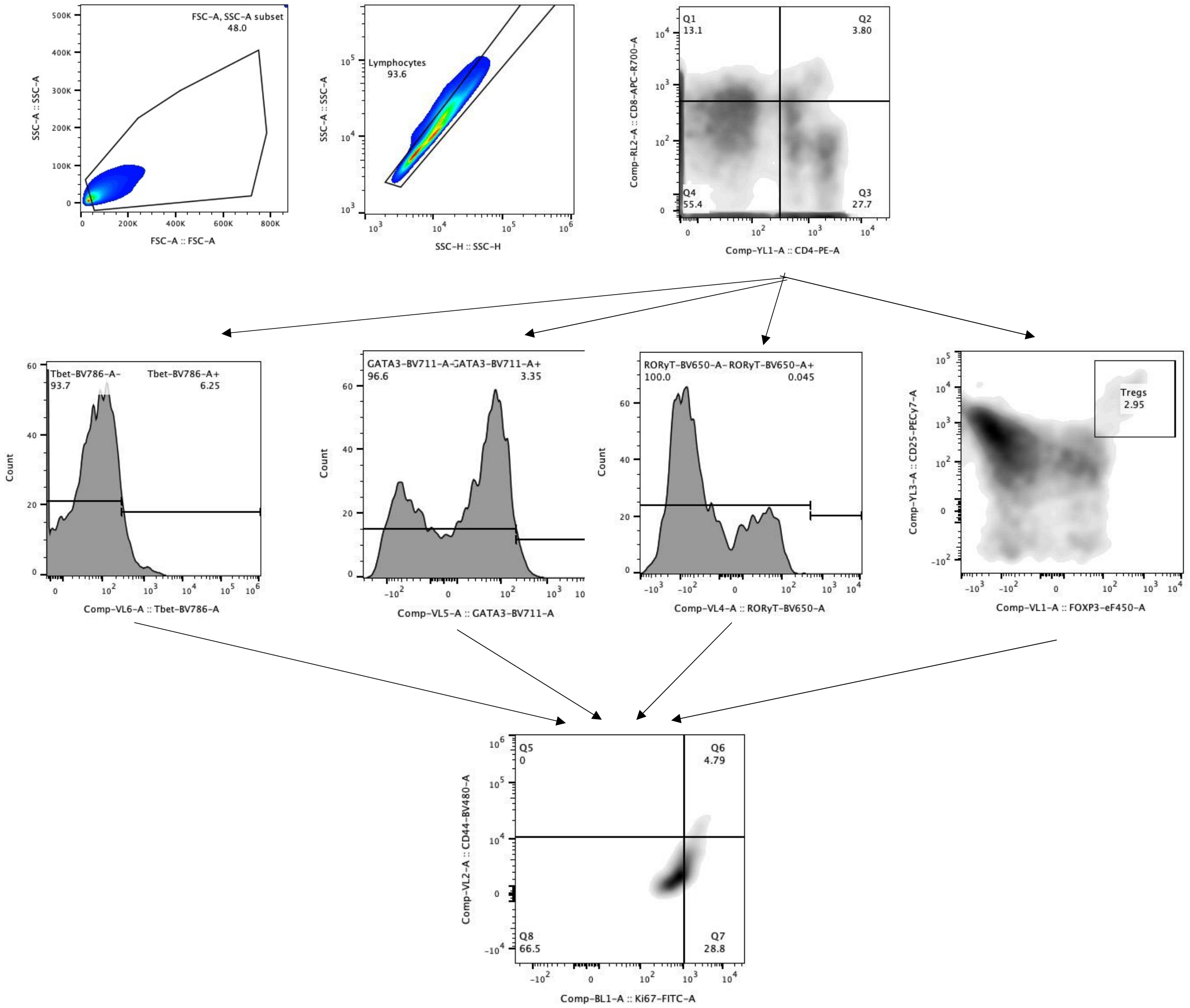

Supplementary Figure 1: Flow cytometry schematic for the analysis of the immune cells for both *in vivo* and *ex vivo* analysis.

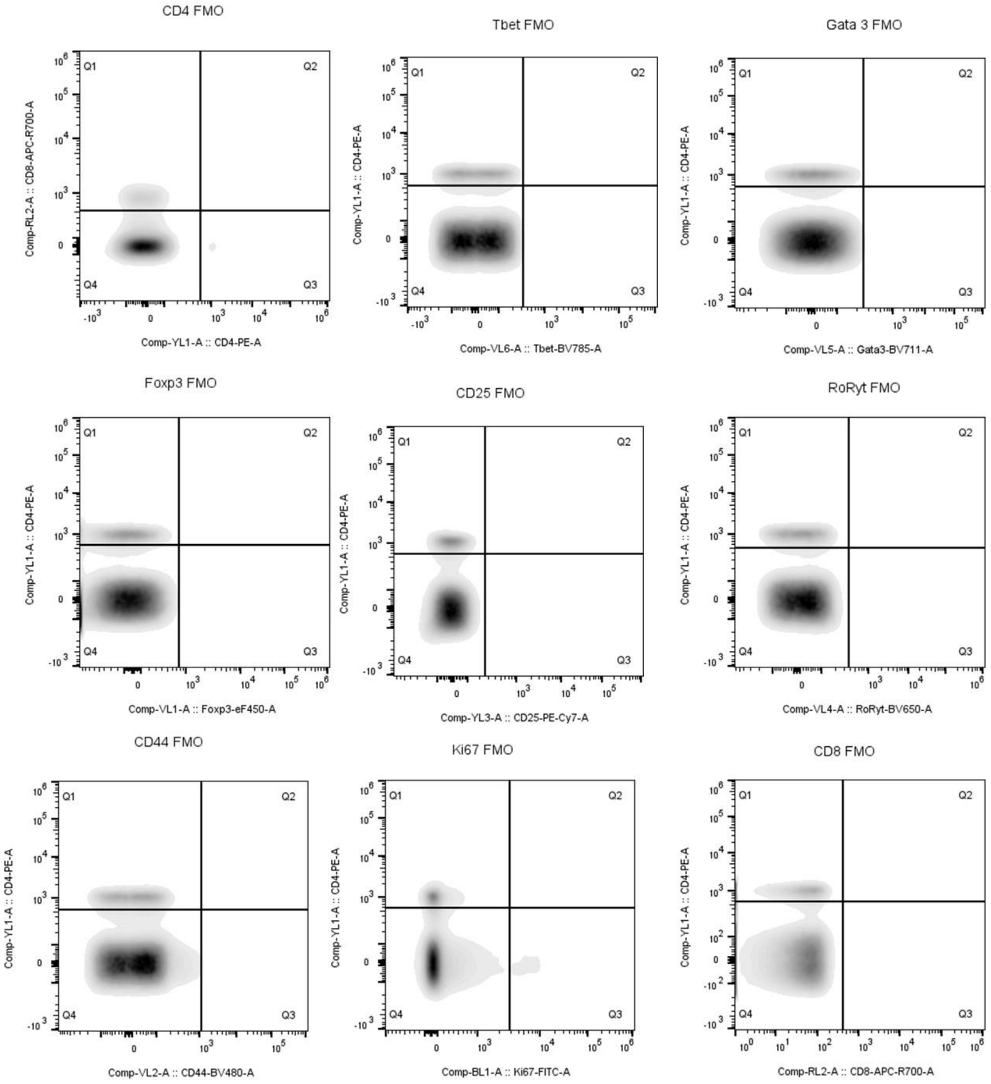

Supplementary Figure 2: Flow cytometry fluorescence minus one (FMO) to determine the negative controls for each of the fluorophore utilized.
